# Functional Assessment of Threshold Parameters for Natural Mineral Water in China: Regulatory Comparison, Hydrogeological Context, Health-Related Evidence, and Label-Based Market Characteristics

**DOI:** 10.3390/foods15152755

**Published:** 2026-08-05

**Authors:** Jiaxuan Shi, Haiyue Yang, Jiaqi Wang, Yi Zhang, Tianjiao Zhang, Jun Wang, Na Zhang

**Affiliations:** 1 Department of Nutrition and Food Hygiene, School of Public Health, Peking University, 38 Xue Yuan Road, Haidian District, Beijing 100191, China; sjx09300731@163.com (J.S.); 13784709242@163.com (H.Y.); 2010108611@stu.pku.edu.cn (Y.Z.); zhangtj428@163.com (T.Z.); 2 China National Center for Food Safety Risk Assessment, 37 Guangqu Road, Chaoyang District, Beijing 100022, China; wangjiaqi@cfsa.net.cn (J.W.); wangjun@cfsa.net.cn (J.W.); 3 Laboratory of Toxicological Research and Risk Assessment for Food Safety, Peking University, 38 Xue Yuan Road, Haidian District, Beijing 100191, China

**Keywords:** drinking natural mineral water, threshold parameters, GB 8537, health-related evidence, hydrogeology, label-based survey

## Abstract

Natural mineral water standards use compositional criteria to define product identity and distinguish natural mineral water from other drinking water categories. China’s National Food Safety Standard—Drinking Natural Mineral Water (GB 8537) specifies seven alternative minimum threshold parameters for this purpose. This study assessed how these parameters function in four dimensions: regulatory identity, hydrogeological resource recognition, health-related interpretation, and label-based market communication. We compared major international regulatory frameworks, qualitatively synthesized health-related evidence, reviewed representative source-scale hydrogeological evidence from China, and analyzed label information from 150 commercial products. China’s system differs from the Codex and European Union frameworks, which emphasize source authenticity, compositional stability, permitted treatment, and disclosure, and from the United States system, which primarily defines mineral water by total dissolved solids. Regional studies showed that mineral water composition reflects site-specific combinations of lithology, geological structure, recharge, groundwater circulation, and water–rock interaction. The same parameter may occur in more than one hydrogeological setting, whereas different parameters within one groundwater system may arise through distinct geochemical processes. Health-related evidence varies substantially and should not be interpreted as establishing consumer health claims. In the label survey, metasilicic acid, TDS, and strontium were the most frequently declared parameters, while zinc and free carbon dioxide were less frequently declared. We propose a functional framework that distinguishes statutory identity, hydrogeological resource, health/safety, sensory/technical, and consumer-communication roles. This framework clarifies why the seven parameters should not be treated as functionally equivalent and provides a basis for refining natural mineral water standards and label communication.

## 1. Introduction

In environmental governance and public health policy, translating complex natural resource characteristics into standardized regulatory criteria consistently represents a major challenge. Policymakers must balance the recognition and sustainable use of geographically distinctive geological resources with the management of potential health risks and the regulation of commercial markets. Natural mineral water, a commercialized natural resource whose identity depends strongly on geological provenance, provides a useful case for examining this policy challenge.

Natural mineral water occupies a distinct regulatory position at the intersection of environmental resources, food governance, and consumer markets. Unlike conventional packaged drinking water, its designation not only depends on potability but also on geological origin, relative compositional stability, and naturally occurring mineral constituents. Under China’s GB 8537, the legally specified compositional criteria used to establish product identity are referred to here as threshold parameters. Regulatory systems assign different functions to such parameters. Regulatory frameworks represented by the Codex Alimentarius Commission (Codex) and the European Union (EU) place greater emphasis on the authenticity of the source, relative compositional constancy, permissible treatment processes, and label disclosure, typically refraining from establishing uniform minimum mineral content thresholds [1,2,3]. In contrast, the United States establishes a TDS level of ≥250 ppm as the foundational definitional criterion for mineral water [4]. In its National Food Safety Standard—Drinking Natural Mineral Water (GB 8537), China constructs the statutory identity framework for natural mineral water by establishing seven threshold parameters: lithium, strontium, zinc, selenium, metasilicic acid, free carbon dioxide, and TDS, requiring that at least one of these parameters be met [5]. In addition to determining statutory identity, these parameters may support hydrogeological resource recognition and may inform health-related interpretation, sensory or technical classification, and market communication [6]. By allowing qualification through any one of seven parameters, this regulatory structure may accommodate a wider range of locally distinctive mineral water resources than a single compositional threshold. It therefore provides a useful basis for examining how mineral water identity criteria operate in a hydrogeologically heterogeneous setting.

The scientific interpretation and practical application of these parameters continue to evolve as geological, toxicological, epidemiological, and market evidence accumulates. At the scientific research level, studies on trace elements—particularly lithium and selenium—have increasingly examined both potential health-related associations and safety concerns, although dose–response relationships and population-specific thresholds remain uncertain [7,8,9]. At the international level, institutional disparities among the WHO, the EU, and the US further suggest that the design of threshold parameters is likely intricately linked to specific hydrogeological contexts, dietary exposure patterns, and hydration habits [2,3,4,10,11]. Recent analytical studies of bottled water products in China have also identified substantial variation in elemental composition, reinforcing the need to distinguish label-declared composition from measured concentrations used in exposure and risk assessments [12].

Previous studies have generally examined individual trace elements, source-water geology, or regulatory requirements separately; consequently, the relationships among statutory identity, hydrogeological resource recognition, health and safety interpretation, and market communication remain insufficiently integrated. We therefore developed a functional framework that distinguished five roles of mineral water parameters: statutory identity determination, hydrogeological resource recognition, health and safety evaluation, sensory and technical characterization, and consumer communication. The study had three objectives. First, we compared the identity, source integrity, treatment, compositional, and labeling provisions of major natural mineral water standards. Second, we evaluated the seven threshold parameters specified in GB 8537 in relation to health-related evidence and China’s principal hydrogeological settings. Third, we assessed how these parameters are communicated on commercial product labels.

## 2. Background and Regulatory Context

### 2.1. Origin, Formation, Production, and Uses of Natural Mineral Water

Natural mineral water originates from a protected underground water table or deposit and acquires its composition through recharge, subsurface circulation, water–rock interaction, mineral dissolution, ion exchange, and, in some settings, geothermal or deep-fault processes [13,14,15,16]. Its defining characteristics are linked not only to potability but also to geological origin, original purity, and relative compositional stability [1,2,5]. Production typically involves abstraction at a recognized spring or borehole outlet, protection of the source, permitted physical treatment when necessary, hygienic bottling, and label disclosure that preserves source traceability and communicates characteristic composition [1,2,3,4,5,17,18].

Natural mineral water is primarily marketed for routine human consumption. In China, medical or physiotherapeutic mineral water resources are considered within geological exploration and resource-evaluation frameworks [19,20]. These resource categories should not be conflated with bottled natural mineral water regulated as a food product or with public drinking water supplied through distribution systems. The present study focuses on drinking natural mineral water; the other two Chinese regulatory domains are included only to clarify differences in regulatory purpose and in the interpretation of concentration criteria.

### 2.2. Regulatory Definitions, Source Integrity, Treatment, and Labeling

The principal regulatory systems use different mechanisms to establish mineral water identity. GB 8537 requires at least one of seven minimum threshold parameters to be met [5]. Codex and EU rules place greater emphasis on underground origin, original purity, compositional stability, source recognition, restricted treatment, and disclosure of analytical composition [1,2,3]. United States regulations define mineral water as water from a protected underground source that contains at least 250 mg/L TDS, has a constant level and relative proportion of minerals and trace elements, and no added minerals. Identity criteria should be distinguished from maximum contaminant limits, which primarily serve safety or sensory-management functions.

Codex and EU definitions permit a single underground water table or deposit to be exploited through one or more natural or borehole outlets, provided that the water remains attributable to the same source system and retains its characteristic composition. GB 8537 requires bottling near the source and declaration of the source point on the label, but it does not establish a general rule for combining waters from distinct source points [5,18]. The permissibility of such combination must therefore be determined from the approved source-appraisal report, production-license documentation, and other applicable administrative requirements.

Under GB 8537-2018, limited physical treatment is only permitted when the basic characteristics and the concentrations of the principal constituents of the source water are not altered. Aeration, decantation, and filtration may be applied to remove unstable constituents. For carbonated products, carbon dioxide originating from the same source may be recovered and reintroduced; food-additive carbon dioxide may be added to produce carbonated natural mineral water; and carbon dioxide may be removed to produce degassed natural mineral water. The use of food additives must comply with the applicable provisions of GB 2760 [5,21]. These provisions permit limited physical stabilization and adjustment of carbonation status while requiring preservation of the natural identity and principal composition of the source water.

For products marketed in China, the GB 8537-specific provisions [5] supplement the general labeling requirements for prepackaged foods under GB 7718 [17]. Labels must identify the natural mineral water source point and the qualifying threshold parameter(s). They must also report ranges for TDS and the principal cations K^+^, Na^+^, Ca^2+^, and Mg^2+^, and include a fluoride statement when fluoride exceeds 1.0 mg/L [5]. A representative compliant label would identify the source point and state the parameter or parameters through which the product qualifies as natural mineral water. It would also provide the required concentration ranges and, where applicable, state that the product “contains fluoride.” These provisions connect regulatory identity with source traceability, analytical composition, and consumer communication [5,17,18].

### 2.3. Scope and Rationale for the Seven Threshold Parameters

The four-dimensional assessment was restricted to lithium, strontium, zinc, selenium, metasilicic acid, free carbon dioxide, and TDS because these seven parameters constitute the alternative minimum identity criteria in GB 8537 [5]. Parameter selection was therefore based on statutory function rather than on the broader chemical, nutritional, or toxicological importance of each constituent. Other constituents—including iron, iodide, fluoride, manganese, nitrate, arsenic, and major ions—may be relevant to sensory quality, technical treatment, nutrition, safety management, or medicinal interpretation, but they do not serve the same regulatory role in the current Chinese identity framework [5,10,22]. Iron primarily functions as a treatment or sensory parameter; iodide was removed from the threshold parameter list in the 2018 revision, and fluoride is managed mainly through maximum limits and labeling requirements. These constituents are included in the regulatory comparison where relevant, whereas the complete health–hydrogeology–market assessment remains focused on the seven statutory threshold parameters.

## 3. Materials and Methods

### 3.1. Inclusion of Relevant International Regulatory/Standard Documents

The inclusion of regulatory and standard documents employed a hybrid approach combining structured database retrieval and supplementary hand-searching. A search conducted in the China National Knowledge Infrastructure (CNKI) using the terms “drinking natural mineral water” and “standard” yielded 371 results. Following the exclusion of the irrelevant literature, one review article concerning mineral water standards was retained. Concurrently, a search in Web of Science using the query (TS = (“standard”) AND TS = (“mineral water”)) identified two relevant review articles, from which nine standard documents pertaining to mineral water threshold parameters were extracted. To reflect contemporary global regulatory practices, relevant standards currently enforced in other countries and regions were supplementarily included, whereas superseded, obsolete, or non-operational documents were excluded. Ultimately, 12 relevant standard documents were included in the analysis. This corpus comprises one international guidance document, two industry standards, and nine national or regional standards. These documents were selected to represent major regulatory models rather than to constitute an exhaustive global inventory.

### 3.2. Selection of Comparator Water Categories, Regulatory Contexts and Concentration Criteria

The analysis was structured around three comparator domains within the Chinese regulatory context: (i) bottled drinking natural mineral water regulated as a food product under GB 8537-2018; (ii) medical or physiotherapeutic mineral water resources considered within the broader geological exploration and resource-evaluation framework, for which GB/T 13727-2016 and GB/T 11615-2010 were used as the principal national standards relevant to natural mineral water and geothermal-resource investigation; and (iii) water intended for human consumption supplied through public systems under GB 5749-2022. The first and third domains were compared directly using legally specified identity, safety, sensory, and general-chemical criteria. The second domain was included as a regulatory-context comparator rather than as a directly equivalent product category because the relevant Chinese documents govern geological exploration and resource evaluation rather than bottled-food regulation, and the regulatory search did not identify a national food safety product standard for bottled therapeutic mineral water that was directly comparable with GB 8537. Accordingly, geological resource-qualification criteria were not interpreted as food safety limits and were not treated as directly comparable concentration thresholds. Reported criteria were classified according to regulatory function as minimum identity thresholds, maximum safety limits, indicator or sensory values, or geological resource-qualification criteria. All seven alternative threshold parameters specified in GB 8537 were retained irrespective of whether corresponding criteria were available in the other two domains. Additional parameters were included when directly comparable maximum or qualification values were specified in the relevant Chinese standards. Where no corresponding criterion was available, the parameter was retained and designated “not specified” or “not directly comparable,” as appropriate.

### 3.3. Qualitative Synthesis Strategy for Health-Related Evidence on Threshold Parameters

A structured literature search was conducted to identify health-related evidence published in English or Chinese between 1 January 2004, and 1 July 2024. The queried databases included PubMed, Embase, MEDLINE, the Cochrane Library, the Chinese Biomedical Literature Service System (SinoMed), the Wanfang Data Knowledge Service Platform, and CNKI. The eligible literature typologies comprised original research articles, narrative reviews, systematic reviews, meta-analyses, as well as relevant institutional reports and standard documents. The supplementary literature was identified through backward reference tracking and manual searches. The specific search terms and search yields for each thematic category are detailed in Table 1. The exclusion criteria were as follows: the literature fundamentally unrelated to the research themes; the articles lacking relevant keywords in the title or abstract; the studies that did not report health outcomes or health-related endpoints associated with the threshold parameters; the literature for which full texts were unavailable; and duplicate publications derived from identical datasets, for which the version containing the most comprehensive data was retained. Data extraction protocols captured the following variables: author(s) and publication year, study design, geographic region, study population, experimental methodology, specific parameter(s) investigated, health outcomes, primary results, and core conclusions. Recognizing the substantial heterogeneity in evidence sources and methodological designs across the various parameters, this study employed a qualitative synthesis of the health evidence rather than quantitative meta-analytic pooling. The quality of evidence was qualitatively appraised with reference to the Evidence-Based Evaluation Guidelines for Food and Health established by the Chinese Nutrition Society, with additional consideration of study design, exposure assessment, outcome relevance, consistency, and directness to drinking water exposure. The health evidence grades used in the present study were derived from the research team’s previous systematic evidence evaluation on the seven threshold parameters and were used in the present study as contextual evidence rather than being re-scored de novo. Therefore, the present manuscript should be interpreted as a functional synthesis and regulatory-contextual analysis rather than an independently reproduced systematic evidence evaluation. Based on study design, methodological quality, exposure assessment, outcome relevance, consistency, and directness to drinking water exposure, the body of evidence was qualitatively stratified into four tiers: excellent, good, moderate, and poor. Subsequently, factoring in consistency, health impacts, population characteristics, and overall applicability, the comprehensive recommendation grades were classified into four levels: A, B, C, and D. To ensure high inter-rater reliability, the screening, inclusion, and exclusion of the literature were conducted independently by two researchers. Disagreements in screening, inclusion, and qualitative interpretation were resolved through discussion and, when necessary, adjudication by a senior researcher.

### 3.4. Collection and Classification of Hydrogeological Source Information in China

Hydrogeological source information was synthesized using multiple source types. The literature was retrieved from CNKI and Wanfang, and relevant official reports and administrative notices published by local governments were included. Brand websites were used only for publicly declared information on product sources. The purpose of this synthesis was to contextualize the relationship between threshold parameters and hydrogeological settings, not to estimate national prevalence or resource abundance. It was a literature-based contextual synthesis rather than an original aquifer-scale investigation; no field sampling, aquifer delineation, hydrochemical modeling, or isotope-based source tracing was conducted. Regional associations were used to interpret the seven threshold parameters and should not be regarded as exhaustive classifications applicable to every mineral water source in China.

### 3.5. Cross-Sectional Survey of Commercial Mineral Water Product Information

The market analysis used a cross-sectional, label-based survey of publicly available product information. The label contents of commercially available, pre-packaged drinking natural mineral waters were systematically collected and collated to extract the declaration methods and indicated numerical values for the seven threshold parameters. A total of 150 valid product label records were included in the analysis after removal of duplicate records and exclusion of products lacking sufficient information on mineral composition. This comprised 123 domestic products, encompassing mainstream and regional brands such as Kunlun Mountain, Nongfu Spring, and Ganten, and 27 international products, covering representative global brands including Evian, Perrier, and San Pellegrino. In accordance with the core regulatory requirements of GB 8537, a standardized data extraction form was developed. This form recorded the specific label claims and reported concentration values for lithium, strontium, zinc, selenium, metasilicic acid, free carbon dioxide, and TDS, while supplementarily capturing data on major cations. All data were independently entered and cross-checked by two researchers, with any missing information explicitly annotated. Because the market survey was based on publicly available label information rather than independent laboratory testing, all threshold patterns identified in this study should be interpreted as label-declared patterns rather than verified hydrochemical compliance categories. In particular, for strontium values of 0.20–0.40 mg/L and metasilicic acid values of 25.0–30.0 mg/L, GB 8537-2018 includes source-water temperature conditions that could not be fully verified from most product labels. Therefore, the label-based analysis was used to examine market communication and declaration practices, not to determine formal regulatory compliance.

## 4. Results

### 4.1. Comparison of Domestic and International Regulatory/Standard Systems

Comparative analysis identified different regulatory functions across the reviewed frameworks. China uses seven alternative minimum threshold parameters as statutory identity criteria and separately regulates maximum concentrations, source integrity, permitted treatment, and labeling requirements [5]. Codex and European Union frameworks emphasize underground origin, original purity, compositional stability, source recognition, restricted treatment, maximum concentrations for selected constituents, and analytical disclosure, without establishing the seven Chinese minimum identity thresholds [1,2,3]. The United States defines mineral water principally by TDS ≥ 250 mg/L and applies additional bottled water quality and labeling requirements [4]. Consistent with the European source- and composition-based approach, the Polish regulation focuses on source assessment, compositional characterization, permitted treatment, maximum concentrations for selected constituents, and chemical or labeling classifications rather than on a separate system of minimum identity thresholds [23]. Accordingly, Table 2 compares the regulatory functions assigned to the seven parameters—including product identity, safety control, analytical characterization, product definition, and labeling classification—rather than treating the reported numerical values as directly equivalent.

### 4.2. Comparison with the Regulatory Position of Medical or Physiotherapeutic Mineral Water Resources and Public Drinking Water in China

Within the Chinese regulatory framework, the same chemical parameter may serve different functions according to the intended use of the water (Table 3). TDS ≥ 1000 mg/L is an alternative identity criterion for drinking natural mineral water under GB 8537, whereas GB 5749 establishes 1000 mg/L as a sensory or general-chemical maximum for public drinking water. Fluoride, selenium, zinc, arsenic, lead, and nitrate are also interpreted differently according to whether the regulatory purpose is product identity, safety management, or sensory acceptability. By contrast, metasilicic acid, free carbon dioxide, lithium, and strontium serve as identity parameters under GB 8537 but are not routine identity criteria for public drinking water [5,22]. Medical or physiotherapeutic mineral water resources are included as a contextual Chinese regulatory domain. Because GB/T 13727-2016 and GB/T 11615-2010 are geological exploration and resource-evaluation standards rather than national food safety product standards, their criteria are not presented as directly comparable food product or public health concentration limits [19,20].

### 4.3. Qualitative Assessment Results of Health Evidence Strength

Based on the research team’s previous systematic evidence evaluation report, this study summarized the health-related associations between the seven threshold parameters and selected health outcomes, with evidence interpreted according to quality, consistency, dose–response clarity, and directness to drinking water exposure (Table 4). Overall, there are manifest disparities in the strength of health evidence across the seven parameters. Selenium exhibits a relatively narrow range between deficiency-related relevance and excess-related risk, making total dietary exposure particularly important when interpreting selenium-containing mineral water. Lithium and strontium show more complex and uncertain dose–response patterns, suggesting that potential health-related associations and risk concerns should be evaluated separately and in population-specific contexts. Evidence regarding the health relevance of metasilicic acid and zinc remains limited; while metasilicic acid possesses a certain baseline of bioavailability, its long-term health benefits lack adequate validation, and zinc intake is predominantly reliant on dietary sources rather than drinking water. The health significance of TDS is primarily contingent upon its specific mineral composition (e.g., calcium and magnesium), whereas elevated TDS levels more frequently impact sensory acceptability rather than posing unequivocal health risks. Finally, free carbon dioxide currently lacks high-quality evidence directly correlating it with specific health outcomes. Given that the existing literature predominantly comprises observational studies, potential confounding factors—including dietary habits, genetic backgrounds, and regional variations—remain difficult to entirely exclude.

### 4.4. Hydrogeological Settings and Representative Regional Evidence for Threshold Parameters in China

Published source-scale studies indicate that the chemical characteristics of natural mineral waters in China are controlled by site-specific combinations of aquifer lithology, geological structures, recharge conditions, groundwater circulation, and water–rock interactions. The associations summarized below should therefore be interpreted as representative regional evidence rather than as a one-to-one national classification of threshold parameters and aquifer types. In the Changbai Mountain volcanic region, hydrochemical and stable-isotope investigations showed that mineral water composition is strongly controlled by rock weathering and the dissolution of feldspar, pyroxene, and olivine. The hydrolysis of these silicate minerals contributes dissolved silica to metasilicic-acid-rich waters [15]. A separate investigation of mineral springs in the same region found that metasilicic-acid springs were associated with rock weathering, whereas metasilicic-acid waters with relatively high carbon dioxide and salinity reflected mixing between shallow groundwater and CO_2_-rich confined thermal groundwater rising along fractures [33]. Comparable lithological control has been reported in northern China. In the Cenozoic basalt aquifer of Zhangbei County, metasilicic acid was attributed mainly to the hydrolysis and leaching of albite, plagioclase, olivine, and other silicate minerals, and stable isotopes indicated recharge predominantly from local summer precipitation [34]. In Chengde, soluble-silica enrichment was controlled by rock-weathering and desilication processes, together with the water-transmitting properties of geological structures; metasilicic-acid mineral waters and geothermal springs commonly occurred at composite structural zones or intersections between major and secondary faults [35]. The Huangshui River catchment in Qinghai provides direct evidence that several threshold parameters may originate through different processes within the same groundwater system. In this catchment, strontium enrichment was attributed mainly to Sr-bearing minerals in gypsum strata and Ca–Sr exchange rather than to carbonate dissolution. Lithium was related to the ancient salt-lake sedimentary environment and the dissolution of lithium-bearing minerals, whereas metasilicic acid was associated with aluminosilicate dissolution. The dissolution of gypsum and halite, together with dedolomitization, contributed to elevated TDS in parts of the catchment [16]. A province-scale investigation in Jiangxi reported that single and composite metasilicic acid mineral waters occurred mainly in intermediate–acid intrusive rocks, weakly metamorphosed rocks, and red clastic rocks. Strontium-, lithium-, and lithium–strontium-type waters occurred mainly in carbonate and red-clastic formations, whereas free-CO_2_-type mineral waters were associated with deep faults that acted as gas-storage or gas-conducting structures [36]. These results illustrate that the same threshold parameter may occur in more than one lithological setting and that geological structures may be as important as aquifer lithology. For selenium, a field investigation in the Northern Daba Mountains examined 62 natural water samples in a region with a high geological selenium background. The waters were predominantly of the Ca·Mg–HCO_3_·SO_4_ type; selenium concentrations were higher in fissure water than in the sampled river water, and eight samples reached the Chinese selenium criterion for drinking natural mineral water [37]. This evidence supports a specific regional association between selenium-rich water and the selenium-rich geological environment of the Northern Daba Mountains, but it does not justify extrapolation to all selenium-bearing mineral waters in China. The source-scale literature identified in the present review did not provide a sufficiently consistent hydrogeological basis for assigning zinc-rich drinking natural mineral water to a specific aquifer or lithological setting at the national level. Therefore, no generalized geological interpretation for zinc was made.

### 4.5. Label-Based Market Survey Results

Label information from 150 commercially available drinking natural mineral water products, including 123 domestic and 27 international products, was analyzed. The survey recorded source location, applicable standard, the seven threshold parameters, TDS, K^+^, Na^+^, Ca^2+^, Mg^2+^, fluoride-related statements, and the provenance of each label image or record. Table 5 summarizes the principal declaration patterns. Among the sampled domestic products, composite label-declared patterns were common, and the seven threshold parameters differed substantially in declaration rates, label-declared value distributions, and apparent market-communication functions. Metasilicic acid, TDS, and strontium were the most frequently declared parameters in the sample and appeared to be commonly used to emphasize source characteristics or compositional identity, whereas selenium-related declarations showed stronger geographical concentration. In contrast, zinc and free carbon dioxide exhibited lower declaration rates. Zinc values were generally close to or below the GB 8537 threshold rather than a regulatory limit, while free CO_2_ declarations were mainly concentrated in naturally carbonated mineral water products, suggesting that both parameters played a relatively limited role in label-based product identification in the sampled market. Furthermore, most sampled domestic products did not present TDS ≥ 1000 mg/L as their apparent label-declared threshold pathway; instead, they articulated compositional profiles through combinations of multiple threshold parameters. The observed differences between domestic and international labels may reflect divergent regulatory logics, labeling requirements, and consumer-communication priorities across different standard frameworks.

## 5. Discussion

Chemical and stable-isotope fingerprinting has distinguished bottled mineral waters by geographic origin with high classification accuracy [38], indicating that source identity is expressed through multivariate composition rather than any single threshold. Consistent with this evidence, the present results support a functional framework in which threshold parameters may contribute to statutory identity, hydrogeological resource recognition, health or safety interpretation, sensory or technical classification, and consumer communication. These functions overlap but are not equivalent.

### 5.1. Functional Positioning of Threshold Parameters Across Diverse Regulatory Systems

Table 2 demonstrates that the principal difference among the reviewed systems lies in the regulatory function assigned to compositional values rather than in a simple hierarchy of stringency. Codex and the European Union emphasize source authenticity, compositional constancy, permitted treatment, official recognition, maximum limits for selected constituents, and analytical composition labeling, while generally refraining from imposing uniform minimum identity thresholds for the seven Chinese parameters [1,2,3]. The United States uses TDS ≥ 250 mg/L as the principal definitional criterion for mineral water [4]. The Polish regulation similarly follows a source- and composition-based model but uses selected values primarily as maximum safety limits or chemical label-classification criteria; it does not establish an alternative minimum-identity system equivalent to GB 8537 [23]. By contrast, GB 8537 requires at least one of seven threshold parameters to satisfy a minimum identity condition and includes source-temperature requirements for borderline strontium and metasilicic-acid concentrations [5]. The revised core difference column in Table 2 therefore distinguishes identity thresholds from analytical requirements, maximum safety limits, and product or label classifications. This structure is compatible with Chinese mineral water resources in which specific constituents may be enriched despite relatively low TDS [39,40,41]. It should not, however, be interpreted as evidence that one regulatory model is universally stricter or scientifically superior to another.

The domestic cross-category comparison further shows that identical numerical values may serve different regulatory purposes. GB 5749 is designed primarily to protect population health, sensory acceptability, and distribution system water quality during long-term public consumption, whereas GB 8537 combines minimum product identity criteria with maximum safety limits, source integrity provisions, permitted-treatment requirements, and labeling obligations [5,22]. Thus, TDS, fluoride, selenium, zinc, and other constituents must be interpreted according to whether the applicable value is an identity threshold, a safety maximum, or a sensory or general-chemical criterion.

Chinese medical or physiotherapeutic mineral water resources occupy a distinct regulatory position. GB/T 13727-2016 provides the geological exploration framework for natural mineral water resources, whereas GB/T 11615-2010 governs the geological exploration of geothermal resources and remains the effective edition before 1 November 2026 [19,20]. Both documents are recommended national standards for resource investigation and evaluation; they are not national food safety product standards, public drinking water standards, or clinical practice guidelines. Their regulatory function is to support the identification, investigation, and appraisal of geological resources rather than to establish directly comparable bottled product identity thresholds, unrestricted public-consumption limits, or authorization for therapeutic claims. Accordingly, Table 3 includes this domain as a contextual comparator and does not numerically rank its resource-evaluation criteria against GB 8537 or GB 5749.

### 5.2. Health Relevance, Evidence Strength, and Uncertainty of Threshold Parameters

Based on the previous systematic evidence evaluation and the qualitative synthesis presented in this review, the current threshold parameters exhibit substantial heterogeneity in the type, strength, and directness of evidence linking them to health outcomes. Evidence for metasilicic acid and strontium suggests possible associations with skeletal, longevity-related, or cardiovascular outcomes, but much of this evidence remains observational, regional, experimental, or otherwise indirect [27,28,42,43,44,45]. Under the GB 8537 threshold framework for metasilicic acid and strontium, drinking water may contribute to total exposure; however, for metasilicic acid concentrations of 25.0–30.0 mg/L and strontium concentrations of 0.20–0.40 mg/L, the standard also includes a source-water temperature condition. Concurrently, certain studies suggest that silicon exposure may be associated with bone-related outcomes [45], indicating that regulatory access thresholds should not be interpreted as health-effect thresholds. Conversely, lithium and selenium possess more complex dose–response relationships and narrower safety margins. While adequate selenium intake is relevant to deficiency prevention and selenium-dependent physiological functions [46,47], selenium has a narrow safety margin, and long-term excessive exposure may increase adverse health risks [48]. For lithium, the exposue ranges potentially associated with benefit or risk remain to be clarified through high-quality research [49,50]. Regarding zinc, although it is an essential trace element, drinking water does not typically constitute its primary dietary source, and excessively high concentrations can negatively impact sensory acceptability [10]. The health relevance of TDS depends more on its constituents than on the aggregate TDS value itself, and free carbon dioxide currently lacks high-quality evidence directly linking it to specific health outcomes. Overall, the existing literature supports the premise that certain threshold parameters possess health relevance or risk-management significance; however, in translating scientific evidence into policy thresholds, policymakers face substantial challenges in navigating scientific uncertainty. When epidemiological evidence remains insufficient to establish universally applicable exposure thresholds, regulatory agencies should consider local dietary exposure, background intake, safety margins, and consumer-communication needs rather than assuming that a uniform threshold can apply across all contexts. Therefore, the grades summarized in Table 4 should be understood as evidence grades for health-related associations derived from the previous systematic evaluation, rather than as direct support for consumer-facing health claims or definitive causal thresholds.

### 5.3. China’s Hydrogeological Interpretation and Implications for Resource Adaptability of Threshold Parameters

The source-scale studies synthesized in Section 4.4 do not support a one-to-one correspondence between individual threshold parameters and single aquifer or lithological types. Instead, mineral water composition reflects site-specific combinations of aquifer lithology, geological structure, recharge, groundwater circulation, and water–rock interaction [15,16,33,34,35,36,37]. Metasilicic acid illustrates this variability: in the Changbai Mountain, Zhangbei, and Chengde studies, enrichment was linked to silicate-mineral weathering or dissolution, while structural pathways and mixing with CO_2_-rich thermal groundwater also influenced some sources [15,33,34,35]. Thus, the occurrence of a given threshold parameter should not be interpreted as evidence of a unique hydrogeological origin.

Different parameters may also arise through distinct processes within a single groundwater system. In the Huangshui River catchment, strontium was related mainly to Sr-bearing minerals in gypsum strata and Ca–Sr exchange, lithium to ancient salt-lake sediments and lithium-bearing minerals, metasilicic acid to aluminosilicate dissolution, and elevated TDS to gypsum and halite dissolution together with dedolomitization [16]. In Jiangxi, metasilicic-acid waters, strontium- and lithium-bearing waters, and free-CO_2_ waters were associated with different lithological and structural settings [36]. Selenium-rich fissure water in the Northern Daba Mountains was documented within a locally selenium-rich geological background [37]. These findings show that the seven regulatory parameters identify compositional outcomes that may result from multiple geological and geochemical pathways.

From a regulatory perspective, the alternative-threshold structure of GB 8537 can accommodate mineral water sources formed under heterogeneous regional conditions without requiring a single national mineralization model. This flexibility should, however, be interpreted cautiously. The available evidence is regional and methodologically heterogeneous and cannot establish the national prevalence of individual mineral water types. Moreover, the literature reviewed here did not provide a sufficiently consistent basis for assigning zinc-rich natural mineral water to a generalized aquifer or lithological setting. Source-specific geological, hydrochemical, and temporal-stability assessment therefore remains necessary, and hydrogeological origin should not be inferred from a label-declared threshold parameter alone.

### 5.4. From Identity Recognition to Consumer Communication: Market Translation of Threshold Parameters and Future Regulatory Directions

Based on public label information from 150 commercially available products, compositional threshold parameters appear not only as regulatory criteria but also as label-based tools for brand narrative, product differentiation, and consumer-facing information [51]. Metasilicic acid, TDS, and strontium were the most frequently declared parameters in the sampled labels and appeared to be used to reinforce source characteristics, compositional identity, and product differentiation. Selenium-related declarations showed geographical concentration in the sampled labels; conversely, zinc and free carbon dioxide were declared less frequently, suggesting more limited roles in label-based identity recognition and market communication within the sampled products. Most sampled domestic products did not present TDS ≥ 1000 mg/L as the apparent label-declared threshold pathway; instead, they commonly presented compositional profiles through combinations of multiple indicators, which is consistent with the GB 8537 logic requiring at least one threshold parameter to meet the specified threshold. Simultaneously, the disparities between domestic and international labels reflect divergent understandings of naturalness, characteristic components, and consumer communication priorities across different regulatory frameworks. Overall, certain threshold parameters simultaneously carry multiple functions, including source-provenance authentication, health-related interpretation, sensory or technical classification, and consumer communication; however, these functions are not always fully aligned. Consequently, future optimizations of standards must further differentiate among authenticity or resource-recognition parameters, health-related parameters, and sensory or technological parameters. Such differentiation could improve the consistency, transparency, and interpretability of label information while maintaining resource recognition and regulatory operability.

## 6. Conclusions and Outlook

This study evaluated the seven GB 8537 threshold parameters through regulatory comparison, qualitative synthesis of health-related evidence, hydrogeological contextualization, and a cross-sectional label survey. Its principal contribution is a functional classification framework that distinguishes statutory identity, hydrogeological resource recognition, health and safety interpretation, sensory and technical classification, and consumer communication.

The seven parameters are not functionally equivalent. The reliability and consistency of the available health-related evidence vary substantially and remain insufficient to interpret statutory identity thresholds as health-effect thresholds. Representative regional studies likewise do not support a simple one-to-one correspondence between individual parameters and a single aquifer type, lithological setting, or formation mechanism. The same parameter may occur under different hydrogeological conditions, while different parameters within one groundwater system may originate through distinct geochemical processes. The frequency and manner in which the parameters are presented on commercial labels also differ considerably. Label declarations should therefore not be used to infer the national prevalence of the corresponding resources or their specific hydrogeological origins.

Taken together, these findings indicate that the seven threshold parameters specified in GB 8537 constitute a composite identity framework with multiple regulatory functions rather than a functionally homogeneous or interchangeable set of criteria. Chinese regulatory practice demonstrates that this framework encompasses statutory product identity, hydrogeological resource recognition, health and safety interpretation, sensory or technical classification, and consumer communication. Although these functions may overlap, they should remain analytically and regulatorily distinct. Future refinement of the standard should further distinguish product identity determination, source-specific geological and hydrochemical appraisal, safety management, sensory or technical classification, and consumer communication. Identity thresholds should not be translated directly into health claims, and label-declared parameters should not be used to infer source genesis or resource distribution. The applicability of this framework to other jurisdictions should be evaluated in relation to local geological conditions, drinking water exposure patterns, dietary structure, risk-management objectives, safety margins, and market conditions.

## Figures and Tables

**Table 1:** Search terms and retrieval yields for the qualitative synthesis of health-related evidence on threshold parameters.

Theme	Search Terms		No. of Documents		Total
	Chinese-Language Search Terms	English-Language Search Terms	Chinese	English	
Qualitative synthesis of health-related evidence on lithium intake from drinking water	(“水” OR “矿泉水” OR “白水” OR “自来水” OR “饮用水”) AND “锂” AND (“健康” OR “疾病” OR “毒理” OR “心血管系统” OR “免疫系统” OR “神经系统” OR “消化系统” OR “泌尿系统” OR “内分泌系统” OR “代谢” OR “骨” OR “骨骼”)	(“water” OR “mineral water” OR “plain water” OR “tap water” OR “drinking water”) AND (“lithium” OR “Li”)AND (“health” OR “disease” OR “disorder” OR “toxicology” OR “toxicity” OR “cardiovascular” OR “heart” OR “immune” OR “immunity” OR “nervous system” OR “mental” OR “digestion” OR “digestive system” OR “urinary system” OR “kidney disease” OR “renal disease” OR “endocrine” OR “metabolism” OR “bone” OR “skeleton”)	2737	39,507	42,244
Qualitative synthesis of health-related evidence on strontium intake from drinking water	(“水” OR “矿泉水” OR “白水” OR “自来水” OR “饮用水”) AND “锶” AND (“健康” OR “疾病” OR “毒理” OR “心血管系统” OR “免疫系统” OR “神经系统” OR “消化系统” OR “泌尿系统” OR “内分泌系统” OR “代谢” OR “骨” OR “骨骼”)	(“water” OR “mineral water” OR “plain water” OR “tap water” OR “drinking water”) AND (“strontium” OR “Sr”)AND (“health” OR “disease” OR “disorder” OR “toxicology” OR “toxicity” OR “cardiovascular” OR “heart” OR “immune” OR “immunity” OR “nervous system” OR “mental” OR “digestion” OR “digestive system” OR “urinary system” OR “kidney disease” OR “renal disease” OR “endocrine” OR “metabolism” OR “bone” OR “skeleton”)	492	1610	2102
Qualitative synthesis of health-related evidence on zinc intake from drinking water	(“水” OR “矿泉水” OR “白水” OR “自来水” OR “饮用水”) AND “锌” AND (“健康” OR “疾病” OR “毒理” OR “心血管系统” OR “免疫系统” OR “神经系统” OR “消化系统” OR “泌尿系统” OR “内分泌系统” OR “代谢” OR “骨” OR “骨骼”)	(“water” OR “mineral water” OR “plain water” OR “tap water” OR “drinking water”) AND (“zinc” OR “Zn”)AND (“health” OR “disease” OR “disorder” OR “toxicology” OR “toxicity” OR “cardiovascular” OR “heart” OR “immune” OR “immunity” OR “nervous system” OR “mental” OR “digestion” OR “digestive system” OR “urinary system” OR “kidney disease” OR “renal disease” OR “endocrine” OR “metabolism” OR “bone” OR “skeleton”)	21,841	961	22,802
Qualitative synthesis of health-related evidence on selenium intake from drinking water	(“水” OR “矿泉水” OR “白水” OR “自来水” OR “饮用水”) AND “硒” AND (“健康” OR “疾病” OR “毒理” OR “心血管系统” OR “免疫系统” OR “神经系统” OR “消化系统” OR “泌尿系统” OR “内分泌系统” OR “代谢” OR “骨” OR “骨骼”)	(“water” OR “mineral water” OR “plain water” OR “tap water” OR “drinking water”) AND “selenium” AND (“health” OR “disease” OR “disorder” OR “toxicology” OR “toxicity” OR “cardiovascular” OR “heart” OR “immune” OR “immunity” OR “nervous system” OR “mental” OR “digestion” OR “digestive system” OR “urinary system” OR “kidney disease” OR “renal disease” OR “endocrine” OR “metabolism” OR “bone” OR “skeleton”)	2357	5928	8285
Qualitative synthesis of health-related evidence on metasilicic acid intake from drinking water	(“水” OR “矿泉水” OR “白水” OR “自来水” OR “饮用水”)AND(“偏硅酸” OR “硅酸” OR “硅”)AND(“健康” OR “疾病” OR “毒理” OR “心血管系统” OR “免疫系统” OR “神经系统” OR “消化系统” OR “泌尿系统” OR “内分泌系统” OR “代谢” OR “骨” OR “骨骼”)	(“water” OR “mineral water” OR “plain water” OR “tap water” OR “drinking water”) AND (“metasilicic acid” OR “partial silicate” OR “silicate”)AND (“health” OR “disease” OR “disorder” OR “toxicology” OR “toxicity” OR “cardiovascular” OR “heart” OR “immune” OR “immunity” OR “nervous system” OR “mental” OR “digestion” OR “digestive system” OR “urinary system” OR “kidney disease” OR “renal disease” OR “endocrine” OR “metabolism” OR “bone” OR “skeleton”)	538	1336	1874
Qualitative synthesis of health-related evidence on free carbon dioxide intake from drinking water	(“水” OR “矿泉水” OR “白水” OR “自来水” OR “饮用水” OR “气泡水”) AND(“游离二氧化碳” OR “游离CO2” OR “气泡水”) AND (“健康” OR “疾病” OR “毒理” OR “心血管系统” OR “免疫系统” OR “神经系统” OR “消化系统” OR “泌尿系统” OR “内分泌系统” OR “代谢” OR “骨” OR “骨骼”)	(“water” OR “mineral water” OR “plain water” OR “tap water” OR “drinking water” OR “Bubble water”) AND (“free carbon dioxide” OR “dissolved free carbon dioxide” OR “free carbon CO2” OR “dissolved free CO2” OR “Bubble water”)AND (“health” OR “disease” OR “disorder” OR “toxicology” OR “toxicity” OR “cardiovascular” OR “heart” OR “immune” OR “immunity” OR “nervous system” OR “mental” OR “digestion” OR “digestive system” OR “urinary system” OR “kidney disease” OR “renal disease” OR “endocrine” OR “metabolism” OR “bone” OR “skeleton”)	123	41	164
Qualitative synthesis of health-related evidence on TDS intake from drinking water	(“水” OR “矿泉水” OR “白水” OR “自来水” OR “饮用水”) AND “水中溶解性总固体” AND (“健康” OR “疾病” OR “毒理” OR “心血管系统” OR “免疫系统” OR “神经系统” OR “消化系统” OR “泌尿系统” OR “内分泌系统” OR “代谢” OR “骨” OR “骨骼”)	(“water” OR “mineral water” OR “plain water” OR “tap water” OR “drinking water”) AND (“Total dissolved solids” OR “TDS” OR “calcium” OR “magnesium”)AND (“health” OR “disease” OR “disorder” OR “toxicology” OR “toxicity” OR “cardiovascular” OR “heart” OR “immune” OR “immunity” OR “nervous system” OR “mental” OR “digestion” OR “digestive system” OR “urinary system” OR “kidney disease” OR “renal disease” OR “endocrine” OR “metabolism” OR “bone” OR “skeleton”)	562	1088	1650

**Table 2:** Comparison of management requirements for threshold parameters in drinking natural mineral water across domestic and international regulatory systems.

Indicators	China (GB 8537-2018) [5]	CODEX/EU [1,2,3]	USA (FDA/IBWA) [4,24]	Poland (Regulation of 31 March 2011) [23]	Core Difference in Management Logic
Lithium (Li)	Threshold Parameter: ≥0.20 mg/L	No lower limit; labeling of composition permitted.	No lower limit; managed as a general constituent.	Included in the full analytical assessment required for qualification; no numerical minimum identity or chemical label criterion is specified.	China: Sets a minimum threshold for characteristic identification. Intl: Typically not a common mineral water identity criterion; where included, as in Poland, it serves analytical characterization rather than product qualification.
Strontium (Sr)	Threshold Parameter: ≥0.20 mg/L; when the concentration is 0.20–0.40 mg/L, source-water temperature should be not lower than 25 °C	No lower limit; usually for labeling only.	No minimum mineral water identity threshold specified.	Included in the full analytical assessment required for qualification; no numerical minimum identity or chemical label criterion is specified.	China: Sets a minimum threshold for characteristic identification. Intl: Stable strontium is generally not used as a common mandatory identity threshold; Poland requires its analysis without assigning an identity value.
Zinc (Zn)	Threshold Parameter: ≥0.20 mg/L	No lower limit; often limited as a sensory indicator	FDA lists zinc at 5.0 mg/L as an aesthetically based allowable level for bottled water, but mineral water is exempt from this allowable level.	No zinc-specific numerical identity, maximum concentration, or chemical label criterion was identified in the supplied regulation.	China: Zinc is used as a threshold parameter for characteristic identification, not as a listed limit indicator under GB 8537-2018. Intl: Zinc is generally managed as a sensory or aesthetic parameter, with mineral water exemptions in some systems.
Selenium (Se)	Threshold Parameter: ≥0.01 mg/L; limit indicator: ≤0.05 mg/L	No lower limit; managed primarily as a health-related limit, with Codex setting a maximum of 0.01 mg/L.	Limit < 0.05 mg/L (FDA).	No minimum identity threshold; maximum concentration: 0.010 mg/L.	China: Balancing both identity floor and safety ceiling. Intl: Consistently viewed as a contaminant requiring strict control; almost no lower limit.
Metasilicic Acid (H_2_SiO_3_)	Threshold Parameter: ≥25.0 mg/L; when the concentration is 25.0–30.0 mg/L, source-water temperature should be not lower than 25 °C	No mandatory lower limit; voluntary labeling.	No lower limit; voluntary labeling.	Included in the full analytical assessment required for qualification; no numerical minimum identity or chemical label criterion is specified.	China: Key characteristic indicator and entry condition. Intl: Viewed as an optional mineral component for labeling, not mandatory for classification. Poland requires analytical determination without establishing an identity or labeling value.
Free Carbon Dioxide (CO_2_)	Threshold Parameter: ≥250 mg/L	No universal lower limit; carbonation is managed through product-type designations such as naturally carbonated, non-carbonated, decarbonated, fortified with carbon dioxide from the source, and carbonated natural mineral water.	No lower limit; managed as sparkling product.	Natural CO_2_ at the source > 250 mg/L qualifies for the corresponding chemical label classification; additional carbonation categories are defined according to CO_2_ origin and degree of saturation.	China: Free CO_2_ is used as one optional quantitative threshold parameter and is also related to carbonated natural mineral water characteristics. Intl: Carbonation is mainly managed through product-type designation, CO_2_ origin, and consumer information.
Total Dissolved Solids (TDS)	Threshold Parameter: ≥1000 mg/L	No uniform lower limit; classification recommended.	Definitional requirement: ≥250 ppm TDS. For mineral water, FDA requires “low mineral content” if TDS is below 500 ppm and “high mineral content” if TDS exceeds 1500 ppm.	Chemical label classifications: ≤50 mg/L, very low mineral content; ≤500 mg/L, low mineral content; 500–1500 mg/L, may be labeled as medium mineral content; >1500 mg/L, high mineral content.	China: Sets high TDS as one optional threshold parameter among seven identity criteria. Intl: TDS is widely used for product definition, grading, or disclosure, with thresholds that differ across systems.

**Table 3:** Comparison of regulatory purposes and selected concentration criteria among drinking natural mineral water, medical or physiotherapeutic mineral water re-sources, and public drinking water.

Parameter or Dimension	Drinking Natural Mineral Water—China, GB 8537 [5]	Medical or Physiotherapeutic Mineral Water Resources—China [19,20]	Public Drinking Water—China, GB 5749 [22]
Regulatory purpose	Bottled-food identity, source integrity, composition, and safety	Geological investigation, resource evaluation, and classification of resources with potential medical or physiotherapeutic use	Safe lifelong consumption through public supply systems
Qualification logic	At least one of seven alternative minimum thresholds, together with source and safety requirements	Assessment under recommended geological standards; not bottled-food identity or public supply safety regulation	Compliance with microbiological, toxicological, sensory, general-chemical, and disinfection requirements
TDS (mg/L)	≥1000 (identity threshold)	Contextual comparison only. Parameter-specific geological resource criteria are not reproduced in this table because their regulatory function is resource identification and qualification rather than bottled-food identity or public drinking water quality control. They are therefore not directly comparable with the concentration criteria established under GB 8537 or GB 5749	≤1000 (sensory/general-chemical maximum)
Metasilicic acid (mg/L)	≥25.0; source temperature ≥25 °C when 25.0–30.0		Not specified
Free CO_2_ (mg/L)	≥250 (identity threshold)		Not specified
Ferrous iron or total iron (mg/L)	No identity minimum in GB 8537		≤0.3 (sensory/general-chemical maximum)
Fluoride (mg/L)	≤1.5; label statement requires above 1.0		≤1.0 (maximum)
Iodide (mg/L)	Not a current identity threshold		Not specified as a routine parameter
Selenium (mg/L)	≥0.01 (identity threshold); ≤0.05 (maximum)		≤0.01 (maximum)
Zinc (mg/L)	≥0.20 (identity threshold)		≤1.0 (sensory/general-chemical maximum)
Lithium (mg/L)	≥0.20 (identity threshold)		Not specified
Strontium (mg/L)	≥0.20; source temperature ≥ 25 °C required when 0.20–0.40 (identity threshold)		Not specified
Arsenic (mg/L)	No identity role; applicable contaminant limits apply		≤0.01 (maximum)
Lead (mg/L)	No identity role; applicable contaminant limits apply		≤0.01 (maximum)
Nitrate as N (mg/L)	Not an identity parameter		≤10 (maximum)

Note: The column for medical or physiotherapeutic mineral water resources represents a geological resource regulatory context rather than a directly equivalent bottled water product category. GB/T 13727-2016 and GB/T 11615-2010 are recommended national standards for geological exploration and resource evaluation; they are not national food safety product standards or public drinking water standards. Parameter-specific criteria contained in these geological documents serve resource identification, investigation, or qualification purposes and are therefore not reproduced or numerically compared with GB 8537 or GB 5749 in this table. In the GB 8537 and GB 5749 columns, “Not specified” means that the cited standard does not establish the parameter as a routine identity, quality, sensory, or maximum-limit criterion. Other applicable regulatory controls may nevertheless exist.

**Table 4:** Summary of report-derived evidence grades and risk characteristics for health-related associations of the seven threshold parameters.

Indicator	Health-Related Association or Relevance	Risk-Related Concern	Evidence Quality and Characteristics	Reported Exposure Nodes/Interpretive Notes	Report-Derived Evidence Grade
Lithium (Li)	May be associated with mental-health outcomes, including suicide-related indicators, while higher exposure during pregnancy has been associated with adverse maternal or offspring outcomes; evidence is therefore health-relevant but directionally complex.	High exposure during pregnancy is associated with fetal development risks.	Primarily observational studies; complex interaction of factors.	Water-concentration evidence: a cohort study suggested that lithium concentrations below 0.031 mg/L were not associated with reduced suicide incidence [25]. Biomarker evidence: pregnancy blood lithium levels should be interpreted separately and should not be directly equated with drinking water concentrations [26].	Good—B
Strontium (Sr)	Observational associations have been reported between strontium in drinking water and longevity or bone-related outcomes, but causality and optimal exposure ranges remain uncertain.	High exposure may increase renal burden (regional studies).	Observational evidence; possible “U-shaped” dose–response.	Longevity-related observational studies reported strontium concentrations of approximately 0.082 mg/L and 0.73 mg/L in specific regional contexts [27,28]. Some regional studies have raised renal-risk concerns at higher exposure levels, but a clear optimal or risk threshold cannot be determined from the available evidence [29,30].	Good—B
Zinc (Zn)	Zinc is an essential trace element, but drinking water is generally not the primary source of zinc intake; water-specific evidence for health benefits remains limited.	High concentration causes sensory discomfort (astringent taste).	Main human source is diet; weak evidence for drinking water contribution.	Sensory upper limit: 3–5 mg/L [10] National Standard lower limit (0.2 mg/L) is far below sensory limit.	Moderate—C (Insufficient water-specific health evidence)
Selenium (Se)	Adequate selenium intake is relevant to deficiency prevention, but the contribution of selenium from drinking mineral water depends on total dietary exposure and has a narrow safety margin.	Narrow safety window; long-term exposure 0.008–0.01 mg/L may increase neurodegenerative risk; >0.05 mg/L increases toxicity risk.	Extremely narrow safety threshold.	Selenium has a narrow safety margin; some studies reported risk-related associations at approximately 0.008–0.010 mg/L [31], while concentrations above 0.05 mg/L are generally associated with greater toxicity concern [32].	Moderate—C
Metasilicic Acid (H_2_SiO_3_)	Some observational and experimental studies suggest potential associations with neurological, cardiovascular, or bone-related outcomes, but high-quality long-term human intervention evidence remains limited.	No clear drinking water exposure risks identified.	Few human intervention studies; short-term RCTs showed no significant effects.	National Standard lower limit (25 mg/L) is a characteristic dose; “Intervention dose” may need to be higher.	Good—B
Free Carbon Dioxide (CO_2_)	No evidence of direct health benefits.	No evidence of direct health risks.	Lack of high-quality human studies.	Not applicable (primarily a sensory/technical indicator).	-
Total Dissolved Solids (TDS)	Any health relevance of TDS is composition-dependent and mainly reflects specific constituents such as calcium and magnesium rather than TDS itself.	Very low TDS water may contribute little to mineral intake; high TDS mainly affects taste and acceptability.	Avoid very low mineral water; high TDS risk is mainly sensory rejection.	Optimal taste: <600 mg/L [10] Sensory rejection: >1000 mg/L. Health risk concern: Very low TDS.	Good—B

Note: Evidence grades were derived from the research team’s previous systematic evidence evaluation report. They indicate the strength of health-related association evidence and should not be interpreted as direct evidence for consumer-facing health claims or definitive causal thresholds.

**Table 5:** Label-declared threshold parameter patterns among commercially available drinking natural mineral water products.

Analysis Dimension	Core Finding	Detailed Explanation/Typical Data
Distribution of label-declared threshold patterns (N = 150)	Composite label-declared patterns were common in the sample	Composite label-declared pattern (≥2 declared indicators reaching GB 8537 threshold values): 95 domestic products and 10 foreign products. The most common declared combination was “metasilicic acid + strontium.” Single label-declared pattern (only one declared indicator reaching the GB 8537 threshold value): 18 domestic products and eight foreign products (17.3%). Incomplete info/Other standards: 10 domestic + 9 foreign (Total 12.7%).
Labeling rate of threshold parameters (Domestic N = 123)	Declaration rates differed substantially among the seven parameters in the sampled domestic labels.	High Declaration Rate (>50%): Metasilicic acid (78.9%), TDS (69.1%), and Strontium (54.5%) Low Declaration Rate (<15%): Lithium (14.6%), Selenium (13.8%) Extremely Low Labeling Rate (<10%): Zinc (6.5%), Free CO_2_ (<5%) These percentages describe the sampled labels and should not be interpreted as national resource prevalence or hydrogeological distribution.
Indicator Content and Market Characteristics (Domestic, N = products with specific label)	Coexistence of threshold-oriented declaration and advantage-oriented communication	Metasilicic acid and strontium: Among products declaring these indicators, many reported values above the GB 8537 thresholds (56.7% and 58.2%) and used these values to highlight compositional advantages. Lithium and selenium: declared values were generally below or close to the GB 8537 thresholds (72.2% and 76.5%), suggesting limited relevance for practical intake contribution based on label-declared concentrations.
TDS and Apparent Label-Declared Threshold Pathway (Domestic, N = products with label)	Most sampled domestic products did not rely on label-declared TDS ≥ 1000 mg/L as their apparent threshold pathway	A vast majority of sampled domestic products with declared TDS values had TDS < 1000 mg/L (95.3%), suggesting that their apparent label-declared threshold pathway was more often based on other indicators, such as metasilicic acid and strontium. This suggests that the single high TDS threshold may have limited practical utility for label-based category differentiation in the sampled products.
Geographical concentration of label declarations (Domestic)	Geographical patterns in the label sample were treated as communication patterns rather than national resource distributions	Selenium-related declarations were concentrated among a limited number of source regions in the collected labels, whereas lithium, zinc, and free-CO_2_ declarations were uncommon. Because the survey was based on market labels rather than a nationally representative hydrogeological sampling design, these patterns were not used to infer aquifer type, formation mechanism, or national resource prevalence.
Domestic vs. International Label Comparison	Labeling focus reflects differences in standard logic	Domestic: Highlights the content of threshold parameters (e.g., values for metasilicic acid, strontium). International: Focuses on TDS values/classification, water source location, and sensory attributes (e.g., sparkling). Rarely actively labels content of lithium, strontium, or selenium.

## Data Availability

The original contributions presented in this study are included in the article. Further inquiries can be directed to the corresponding author.
